# Changes to local area public sector spending and food purchasing in England: a longitudinal ecological study

**DOI:** 10.1136/bmjnph-2021-000346

**Published:** 2022-03-04

**Authors:** Rosemary H Jenkins, Eszter P Vamos, David Taylor-Robinson, Kate E Mason, Anthony A Laverty

**Affiliations:** 1 Public Health Policy Evaluation Unit, Imperial College London, London, UK; 2 Department of Public Health, Policy and Systems, University of Liverpool, Liverpool, UK

**Keywords:** dietary patterns

## Abstract

**Objectives:**

Changes in public sector service spending may influence food consumption. We make use of changing local authority (LA) expenditure in England to assess impacts on food purchasing. We examine total LA service spending and explore two potential pathways: highways and transport spending which may affect access to food; and housing service expenditure which may affect household resources available to purchase foods.

**Design:**

Longitudinal panel survey at the LA level (2008–2015) using fixed effects linear regression.

**Setting:**

324 LAs in England.

**Main exposure:**

Expenditure per capita on total LA services, highways and transport services, and housing services.

**Main outcome measures:**

LA area estimates of purchasing of fresh fruits and vegetables, high in fat, sugar and salt (HFSS) foods, and takeaways at home, expressed as a percentage of total food and drink expenditure.

**Results:**

Total LA service spending decreased by 17% on average between 2008 and 2015. A 10% decrease in total LA spending was associated with a 0.071 percentage point decrease in HFSS (95% CI −0.093 to –0.050) and a 0.015 percentage point increase in takeaways (95% CI 0.006 to 0.024). A 10% decrease in highways and transport expenditure was associated with a 0.006 percentage point decrease in fruit and vegetable purchasing (95% CI −0.009 to –0.002) and a 0.006 percentage point increase in takeaway purchasing (95% CI 0.001 to 0.010). These associations were seen in urban areas only when analyses were stratified by rural/urban area status. A 0.006 percentage point decrease in HFSS purchasing was also seen with a 10% decrease in housing expenditure (95% CI −0.010 to –0.002).

**Conclusion:**

Changes in LA spending may have impacts on food purchasing which are evident at the area level. This suggests that in addition to more prominent impacts such as foodbank use, austerity measures may have mixed impacts on food purchasing behaviours among the wider population. Individual-level research is needed to further elucidate these relationships.

**Key messages**

**What is already known on this topic**

- Studies suggest that reductions in local authority spending as a result of austerity policies may have impacted health and widened health inequalities in the UK. Research on the impact of austerity policies on food consumption is limited to food insecurity and foodbank use and tends to focus on impacts of welfare reform rather than changes to public sector spending. Impacts of changes to public sector spending on diets have yet to be examined. This study is the first to investigate the impact of reductions in local authority service spending on food purchasing.

**What this study adds**

- This study provides evidence that changes in local authority spending were associated with changes in purchasing of fruits and vegetables, foods high in fat, sugar and salt, and takeaways. We found that a decrease in total local authority service spending was associated with a small decrease in purchasing of foods high in fat, sugar and salt as a percentage of total food and drink purchases and a small increase in takeaway purchases as a percentage of total food and drink purchases. Reductions in highways and transport and housing spending individually had impacts on food purchases, elucidating some potential pathways of these impacts.

**How this study might affect research, practice or policy**

- This study shows that further research is needed to examine the impacts of austerity policies on diets, particularly at the individual level and regarding mechanisms through which impacts may occur. Our study suggests that decreases in local authority service expenditure may impact food consumption, which may lead to health impacts. Thus, policy-makers and healthcare workers should consider the diet and health impacts of reductions to local authority budgets and austerity policies.

## Introduction

Public sector spending can impact individuals’ health. Government spending, including pensions and social care, family payments, and housing allowances and subsidies, was associated with better health outcomes in a cross-national study.^1^ However, in 2010, the UK implemented austerity policies, which involved national welfare reform including a new benefits system, increased conditionality, and changes to benefit eligibility and amounts households could receive.^2^ Austerity policies also involved reductions in funding for local authorities (LAs). LAs are responsible for a range of local services.^3^ Furthermore, they have a statutory duty to improve the well-being of the local community and can be important in mitigating health inequalities.^4 5^ Austerity policies led to decreased expenditure on public service provision; these reductions varied across LAs and were implemented at a time of widening health inequalities and increasing food prices.^6–8^ The Marmot Review: Ten Years On has hypothesised that cuts to LA service expenditure have harmed health and widened health inequalities.^9^ Research suggests that decreases in funding for LAs are associated with decreases in life expectancy,^10^ highlighting potential impacts of lower LA service spending on health.

**Figure 1:**
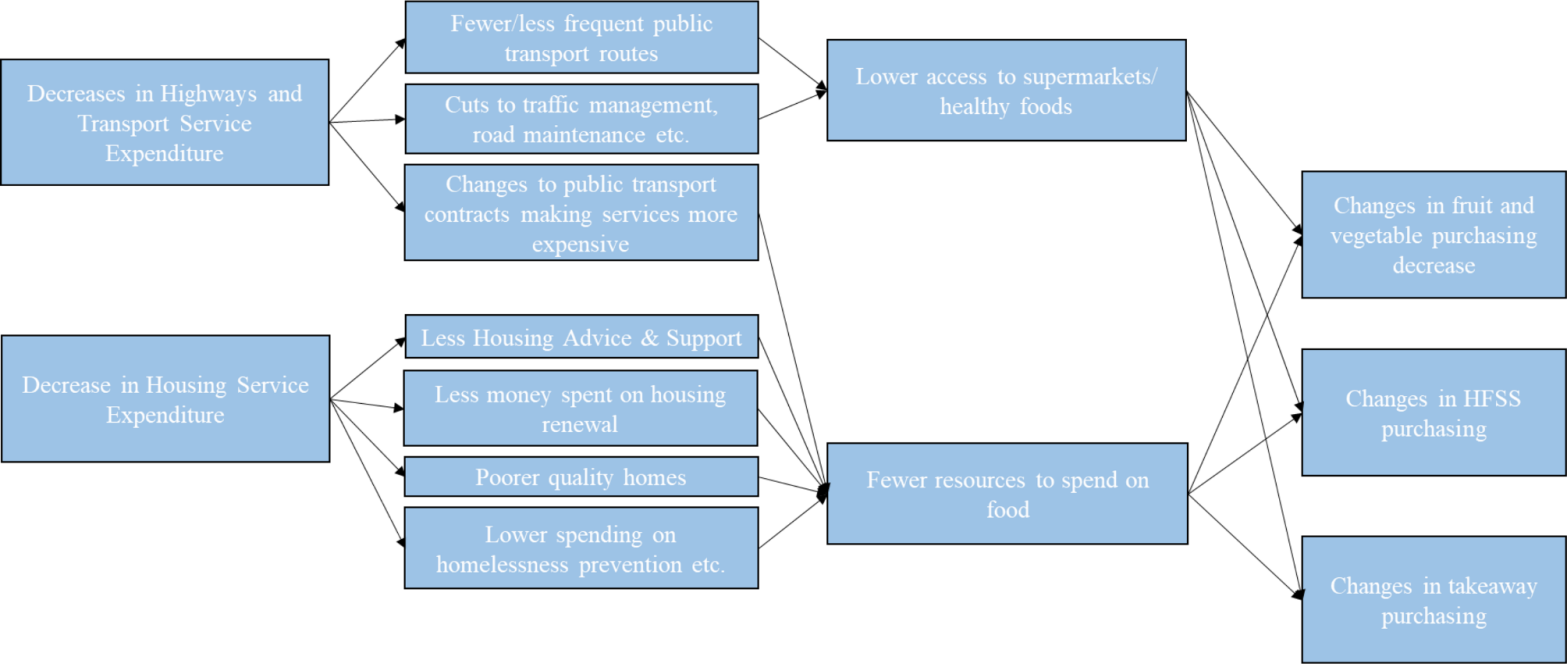
Potential pathways from LA expenditure to food purchasing. HFSS, high in fat, sugar and salt; LA, local authority.

One way LA service spending may impact health is via impacts on diets, but this is under-explored as existing research on impacts of austerity policies has focused predominantly on welfare reform and the use of foodbanks.^11^ However, small shifts in food consumption may have large population health impacts and thus impacts on diets may be important factors in impacts on health and health inequalities.^12^ Although there may be many mechanisms through which national and overall local-level service spending may influence diets, we propose two specific pathways through which we hypothesise LA spending changes may affect household food purchasing (figure 1).

First, we hypothesise that decreases in highways and transport spending may influence access to a healthy diet. This LA budget line includes highways and road maintenance and public transport (including concessions, paying public transport operators and public transport coordination).^3^ Components of highways and transport spending have been cut differentially, with the greatest decrease in traffic management and smaller decreases in public transport.^8^ Only spending on parking services increased.^8^ Therefore, reductions in highways and transport spending may impact people’s access to shops, especially through reductions in public transport. US evidence suggests that public transport plays an important role in people accessing healthy food and supermarkets and that a lack of access to public transport may lead to increased associations between local fast food environment and body weight.^13–15^ Thus, cuts to LA spending on transport may affect provision of public transport, which may in turn lead to reduced access to food shops. Supermarket access is important in access to healthy food.^16 17^ A lack of access to large supermarkets may lead to individuals shopping at smaller, local supermarkets, which tend to have lower availability and quality of healthful foods.^18^ Therefore, reductions in LA transport spending may lead to decreased access to healthy food and increased reliance on unhealthy fast food.

The second pathway we propose is through reductions in LA housing service expenditure. LA housing service expenditure includes spending on housing advances, private sector housing renewal and homelessness including temporary accommodation.^3^ Some areas of funding within housing services decreased more than others, particularly services providing advice and support for vulnerable groups.^8^ These reductions may mean that individuals may bear more of the housing costs themselves. Housing costs are a large expense in household budgets, meaning higher housing costs can limit available household resources and may lead to trade-offs between housing costs and paying for other items, including healthy food.^19 20^ Decreases in LA housing service expenditure may therefore lead to a reduction in household resources to spend on food.^21^ As foods high in fat, sugar and salt (HFSS) are typically cheaper and therefore more affordable than healthier foods such as fruits and vegetables, this may have negative impacts on both health and inequalities.^22^

In this study, we made use of the natural experiment of changing LA expenditure in England to assess impacts on patterns of food purchasing likely to impact population health. We aimed to investigate the association between reductions in total LA service spending on food purchasing. We further assess the association with specific expenditure on highways and transport spending, and housing services spending, aligned to our hypotheses described above.

## Methods

### Exposure variables

The Ministry of Housing, Communities & Local Government (MHCLG) reports LA expenditure on various public services each year, compiled in and available from the Place-Based Longitudinal Data Resource.^23–29^ We used data on gross spending per capita for 324 lower tier LAs in England (excluding the Isles of Scilly and the City of London). We calculated the sum of gross LA spending excluding court services, public health services (both not available for all years), education services (due to changes from LA schools to academy provision), police services and fire and rescue services (provided by separate authorities and funded separately by specific central government grants and locally levied precepts).^30^ We also used specific service expenditure budget lines: highways and transport service expenditure and housing service expenditure.^3 23 25^ All data were adjusted for inflation using the Consumer Price Index, pegged to 2015. We log transformed our exposure variables to account for diminishing returns on investment.

### Outcome variables

The Living Costs and Food Survey is an annual, cross-sectional, nationally representative survey of household expenditure in the UK.^31^ James *et al* used spatial microsimulation methods on these data to derive estimates of weekly expenditure per person per LA for 96 categories of food eaten both in the home and out of the home.^32^ To do this, they matched individual data to the LA population, taking employment, ethnicity, population and income characteristics into account through a series of constraint tables, such that the estimates are representative of the LA population.^32^ They also accounted for regional differences in food prices.^32^

Our primary outcomes were spending on each of fresh fruit and vegetables at home, HFSS foods at home, and takeaways by individuals as a percentage of all food and drink purchasing, estimated at the LA level. These food categories were chosen as important indicators of healthy and unhealthy food purchasing, respectively, and we examined them as a percentage of food and drink purchasing to account for differences in overall household food and drink expenditure. The full list of product categories included in these variables can be found in online supplemental appendix 1; the three variables were mutually exclusive and did not contain any of the same food items. We calculated the sum of purchasing of HFSS foods following the UK government definition.^33^ The year 2009 was excluded from these outcomes as data were not available. We also examined as secondary outcomes the absolute amounts (in £) spent per year on each category.

10.1136/bmjnph-2021-000346.supp1 Supplementary data

### Covariates

We included LA gross disposable household income (GDHI), unemployment rate and expenditure on other LA services as our time-variant covariates in the model because we identified these as potential LA-level confounders. GDHI is an area-level measure of the amount of money individuals have available for spending or saving after receipt of benefits and payment of taxes.^34^ It can be used as a measure of local level economic conditions as it measures local economic diversity and social welfare.^34^ We obtained GDHI data (which we adjusted for inflation) and model-based estimates of LA unemployment rates from the Office for National Statistics (ONS).^34 35^ ONS calculated the unemployment rates using estimates from the Labour Force Survey and the claimant count of unemployment benefit recipients.^35^ Finally, we summed expenditure on other LA spending categories to adjust for spending on other services in the analyses of highways and transport and housing service expenditure.

We also undertook analyses to assess effect modification. We stratified by the time-invariant characteristics of Index of Multiple Deprivation (IMD), rural/urban area status and level of reductions in working age benefits, identified a priori as potential effect modifiers. We also obtained IMD 2015 from MHCLG.^36^ We used the average rank of LA IMD to make quintiles of relative deprivation. Whether an LA was predominantly rural, predominantly urban, or urban with significant rural was included, as this may affect both council expenditure and individual purchasing.^37^ These data were obtained from the ONS Open Geoportal based on the 2011 rural–urban LA classification.^38^ Welfare reform is another key aspect of austerity policies. To account for this, we stratified by quartiles of reductions in working age benefit by LA, using the Uneven Impact of Welfare Reform dataset (on request from the authors) which estimates the cumulative decreases in benefits for working age people due to welfare reforms between 2010 and 2015 for each LA.^39^

### Analyses

We descriptively examined total LA expenditure, highways and transport expenditure, and housing service expenditure in 2008 and 2015, including differences by the covariates and potential effect modifiers. We also calculated percentage change between 2008 and 2015. We also tabulated average food purchasing of fruit and vegetables, HFSS foods, and takeaways in terms of both money spent and purchasing as a percentage of total food and drink expenditure. We assessed the relationship between change in LA service spending and change in food purchasing using scatter plots.

We used a linear fixed effects panel regression approach for the main analysis. Panel regression models are used to investigate units of observation (in this case, LAs) to be followed over time while taking into account clustering of data over time.^40^ Fixed effects linear regression allows examination of associations between exposures and outcomes that vary over time (2008–2015) while controlling for time-invariant, unobserved factors. Cluster-robust standard errors were used to adjust for potential heteroskedasticity and autocorrelation.^41^ We used time dummy variables to account for England-wide time effects. Given that certain LA characteristics including employment, ethnicity, population and income characteristics/counts were taken into account to some extent in the development of our outcome data,^32^ we present unadjusted models. We also present models adjusting for time-variant factors GDHI and unemployment rate. LA expenditure on other services was adjusted for in our models with highways and transport and housing service spending. We subsequently stratified all our adjusted models for IMD, rural/urban area status, and extent of reductions to working age benefits to test our a priori hypotheses regarding effect modifiers. As exposure data were log-transformed, the coefficients presented in the tables are interpreted as absolute changes for a 10% decrease in LA service spending. This level of decrease in the exposure was chosen as it represented the scale of the observed reductions in service spending, which although on average were smaller than 10% each year, accumulated over time to represent reductions of more than 10%.

We also conducted some additional analyses. First, we also conducted a negative exposure control analysis using cultural spending as the exposure, as we would not expect this exposure to influence food purchasing. This assessed unmeasured confounding in the model – no statistically significant association suggests limited residual confounding such that a causal interpretation of the primary association is more plausible. Second, we conducted a supplementary analysis of the adjusted models with time lags for one and 2 years in order to examine medium term effects. Finally, bootstrapping methods were employed for the unadjusted and adjusted models. The bootstrapping methods used involved making use of different samples of the LAs included in this study, in order to assess the role of LA sampling on the results and ensure results were not affected by a small number of LAs. We utilised bootstrapping methods with 1000 reps.

## Results

**Figure 2:**
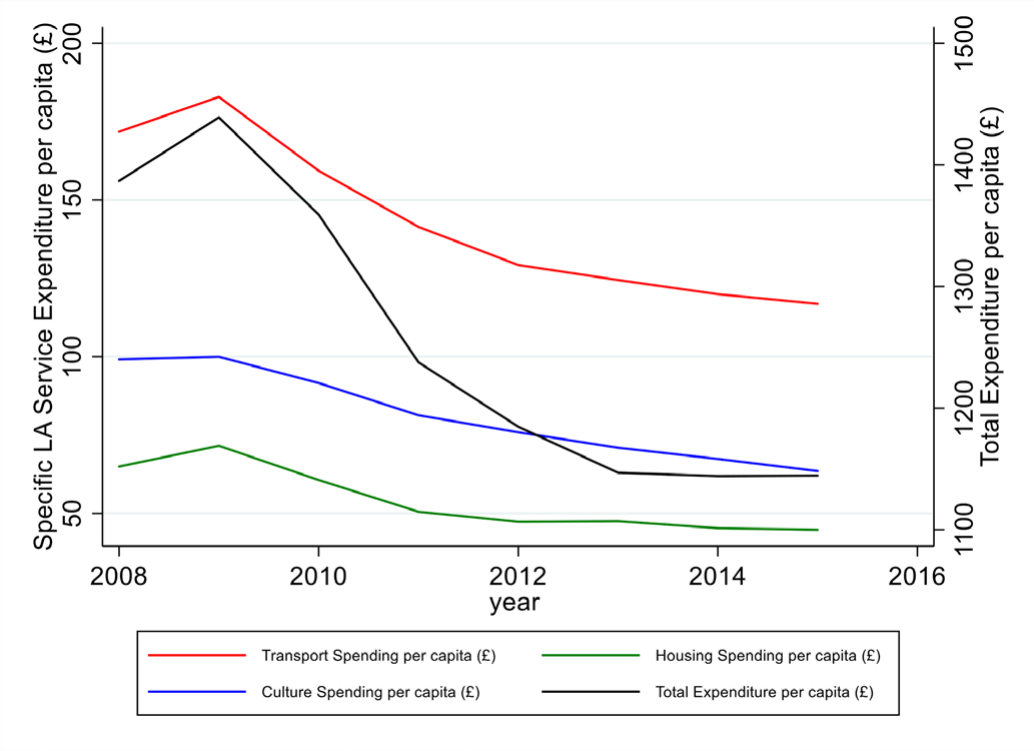
Change in local authority spending over time. LA, local authority.

Total LA expenditure decreased by 17% on average between 2008 and 2015 (table 1). Greater decreases were seen in areas which were more deprived, were urban, had higher unemployment rates and had greater reductions in working age benefits. Highways and transport spending and housing spending decreased by an average of 32% and 35% respectively over the same period. Changes in specific areas of LA expenditure are shown in figure 2, which shows that LA spending increased between 2008 and 2010 and then decreased.

**Table 1:** Average LA expenditure per year and change between 2008 and 2015

	Total LA expenditure per capita (£) per year: mean (SD)		% change	Highways and transport LA expenditure per capita (£) per year: mean (SD)		% change	Housing services LA expenditure per capita (£) per year: mean (SD)		% change
	2008	2015		2008	2015		2008	2015	
**Total**	1386.9 (366.8)	1144.5 (220.1)	−17	171.8 (112.7)	116.9 (71.9)	−32	64.9 (65.4)	42.2 (45.2)	−35
Region									
North East	1610.4 (253.4)	1300.3 (159.1)	−19	159.6 (21.8)	151.8 (44.0)	−5	59.2 (27.0)	34.4 (15.1)	−42
North West	1429.8 (269.3)	1195.1 (185.4)	−16	163.9 (43.9)	107.6 (31.7)	−34	60.1 (41.1)	31.5 (15.2)	−48
Yorkshire and the Humber	1305.8 (163.9)	1115.9 (125.6)	−15	142.7 (30.3)	105.5 (23.2)	−26	63.2 (19.4)	33.0 (11.2)	−48
East Midlands	1198.9 (169.9)	1026.1 (114.3)	−14	132.3 (29.5)	80.8 (28.3)	−39%	31.9 (34.9)	26.2 (13.4)	−18
West Midlands	1259.6 (188.7)	1970.8 (131.7)	−15	127.0 (27.0)	88.3 (23.2)	−31	43.1 (26.3)	40.0 (54.9)	−7
London	2145.2 (574.3)	1540.1 (349.0)	−28	486.3 (79.8)	313.3 (41.0)	−36	194.7 (117.0)	139.6 (78.9)	−28
South West	1264.2 (148.5)	1108.2 (142.6)	−12	142.7 (32.3)	99.0 (28.5)	−31	60.0 (26.4)	31.2 (15.5)	−48
East of England	1321.7 (165.5)	1070.7 (108.5)	−19	137.8 (36.5)	89.1 (20.6)	−49	45.6 (27.5)	35.4 (16.4)	−22
South East	1265.9 (182.2)	1082.3 (128.1)	−15	120.7 (34.8)	89.3 (18.4)	−26	53.1 (43.6)	39.9 (20.5)	−25
GDHI*									
Lowest quartile (GDHI <£16 700)	1358.3 (224.7)	1138.3 (167.1)	−16	141.0 (52.5)	98.6 (38.8)	−30	54.8 (31.0)	36.2 (18.6)	−34
Second quartile (GDHI £16 700–£18 461)	1302.9 (288.5)	1138.3 (245.1)	−13	148.6 (77.3)	115.9 (77.6)	−22	52.7 (42.5)	47.8 (57.4)	−9
Third quartile (GDHI £18 461–£20 725)	1489.5 (504.0)	1177.2 (243.1)	−21	224.9 (165.4)	139.2 (94.5)	−38	87.2 (96.5)	56.0 (54.5)	−36
Highest quartile (GDHI>£20 725)	1356.6 (306.3)	1122.5 (211.2)	−17	155.1 (73.4)	109.0 (54.4)	−30	57.5 (50.3)	38.2 (38.8)	−34
Unemployment rate									
Lowest quartile (<4.55% unemployed)	1206.6 (141.0)	1058.9 (123.6)	−12	132.4 (57.9)	91.4 (28.0)	−32	41.8 (18.7)	31.2 (23.8)	−25
Second quartile (4.55%–5.90% unemployed)	1326.3 (172.6)	1163.6 (218.7)	−12	158.4 (81.8)	125.7 (86.3)	−21	50.9 (46.5)	47.2 (40.4)	−7
Third quartile (5.90%–7.74% unemployed)	1547.0 (472.0)	1334.8 (292.2)	−14	214.1 (150.6)	179.8 (99.7)	−16	85.1 (77.4)	83.7 (78.7)	−2
Highest quartile (>7.74% unemployed)	1882.8 (422.1)	1330.5 (165.7)	−29%	264.1 (149.9)	135.9 (59.2)	−49	147.1 (105.3)	49.9 (57.4)	−66
Level of reductions to working age benefits per person per year**†**									
Lowest quartile (<£321.5)	1206.5 (159.1)	1049.5 (126.8)	−13	129.5 (63.1)	93.8 (41.7)	−28	40.0 (20.3)	30.3 (17.3)	−25
Second quartile (£321.5–£403)	1269.8 (249.3)	1083.5 (155.8)	−15	161.1 (100.3)	106.5 (64.5)	−34	48.5 (46.6)	35.4 (32.9)	−10
Third quartile (£403–£479)	1458.1 (401.7)	1168.2 (215.4)	−20	187.8 (122.5)	126.7 (78.6)	−33	71.3 (66.2)	49.8 (42.1)	−30
Highest quartile (>£479)	1586.1 (370.3)	1260.9 (249.9)	−21	202.3 (121.3)	138.5 (82.8)	−31	97.5 (79.2)	60.3 (59.5)	−38
IMD**‡**									
1 (most deprived)	1742.6 (504.4)	1334.3 (284.6)	−23	236.6 (154.1)	154.0 (91.9)	−35	119.1 (102.7)	72.0 (72.4)	−40
†2	1471.2 (373.3)	1197.7 (244.1)	−19	185.3 (122.2)	130.2 (83.6)	−30	77.1 (59.1)	55.5 (53.6)	−28
‡3	1296.7 (182.1)	1093.1 (120.2)	−16	154.9 (81.5)	103.3 (58.3)	−33	52.21 (48.5)	37.0 (24.4)	−29
4	1222.3 (171.8)	1054.4 (126.6)	−14	153.0 (89.5)	103.2 (55.5)	−33	38.4 (19.3)	28.9 (16.2)	−24
5 (least deprived)	1207.5 (155.8)	1046.4 (123.3)	−13	131.0 (66.6)	94.5 (43.9)	−28	38.8 (18.4)	30.9 (17.0)	−20
Rural/urban									
Predominantly urban	1518.8 (434.5)	1213.1 (256.6)	−20	205.5 (140.0)	138.7 (88.2)	−33	83.5 (80.8)	56.8 (54.9)	−32
Urban with significant rural	1243.1 (134.6)	1076.3 (126.6)	−13	139.4 (30.0)	87.1 (18.4)	−38	40.8 (24.8)	30.3 (11.3)	−26
Predominantly rural	1209.4 (134.5)	1048.4 (110.6)	−13	130.0 (30.8)	91.1 (27.2)	−30	42.2 (21.2)	29.5 (28.3)	−30

*GDHI per person per year, categorised into four quartiles.

†We stratified by quartiles of reductions in working age benefit by LA, using a dataset estimating the cumulative decreases in benefits for working age people due to welfare reforms between 2010 and 2015 for each LA.

‡IMD represents relative deprivation of LAs, categorised into quintiles.

GDHI, Gross Disposable Household Income; IMD, Index of Multiple Deprivation; LA, local authority.

On average across the whole study period, individuals spent £201 on fresh fruit and vegetables at home per year, which made up 11% of their overall food and drink purchasing (table 2). They spent £543 on HFSS foods per year, making up 29% of food budgets, and £103 on takeaways per year, only 6% of food and drink purchasing. Purchasing of fruit and vegetables as a percentage of overall food and drink purchases did not differ substantially by the sociodemographic variables. Money spent on fruit and vegetables, HFSS and takeaways each was greater in LAs with lower unemployment rates or experiencing a lesser reduction in working age benefits. Fruit and vegetables and HFSS purchasing were greater in rural areas, but takeaway purchases were higher in urban areas. Money spent on fruit and vegetables and HFSS was lower by deprivation, but money spent on takeaways remained at similar levels. Food purchasing did not change considerably over time, including when stratified by deprivation or region (data not shown).

**Table 2:** Purchasing of foods per year, averaged across 2008–2015 by sociodemographic variables

	Fruit and vegetables		HFSS		Takeaways	
	£ per year	% of total food and drinks	£ per year	% of total food and drinks	£ per year	% of total food and drinks
**Total**	200.6 (14.8)	10.7 (0.3)	542.6 (38.8)	28.9 (1.1)	102.8 (7.7)	5.5 (0.5)
Region						
North East	191.5 (11.1)	10.6 (0.2)	539.8 (25.3)	29.8 (0.5)	98.8 (3.6)	5.5 (0.3)
North West	193.8 (13.6)	10.7 (0.2)	535.3 (33.1)	29.5 (0.5)	99.0 (3.7)	5.5 (0.3)
Yorkshire and the Humber	192.8 (13.4)	10.7 (0.3)	532.3 (30.0)	29.5 (0.5)	97.5 (3.8)	5.4 (0.3)
East Midlands	199.6 (13.1)	10.7 (0.3)	5486 (32.8)	29.4 (0.6)	100.6 (4.0)	5.4 (0.4)
West Midlands	198.4 (14.5)	10.7 (0.3)	541.1 (37.0)	29.3 (0.5)	100.1 (4.5)	5.4 (0.3)
London	197.4 (16.4)	10.6 (0.5)	487.0 (45.5)	26.3 (1.0)	119.6 (8.4)	6.5 (0.5)
South West	207.0 (13.0)	10.7 (0.3)	565.4 (25.5)	29.4 (0.5)	100.0 (5.0)	5.2 (0.4)
East of England	202.3 (13.5)	10.7 (0.3)	549.2 (30.9)	29.0 (0.7)	102.0 (5.0)	5.4 (0.4)
South East	206.9 (13.9)	10.7 (0.3)	557.2 (30.3)	29.8 (0.7)	104.2 (4.8)	5.4 (0.4)
GDHI*						
Lowest quartile (GDHI<£16 700)	199.2 (13.3)	10.7 (0.3)	545.2 (30.9)	29.2 (0.8)	101.2 (5.7)	5.4 (0.4)
Second quartile (GDHI £16 700–£18 461)	201.4 (15.4)	10.7 (0.3)	544.8 (39.7)	28.9 (1.1)	103.1 (8.1)	5.5 (0.6)
Third quartile (GDHI £18 461–£20 725)	201.6 (15.6)	10.7 (0.3)	534.0 (47.3)	28.4 (1.4)	106.2 (9.8)	5.7 (0.6)
Highest quartile (GDHI>£20 725)	200.0 (14.6)	10.7 (0.3)	546.3 (34.1)	29.3 (0.8)	100.7 (5.2)	5.4 (0.3)
Unemployment rate						
Lowest quartile (<4.55% unemployed)	211.1 (12.3)	10.7 (0.3)	567.3 (23.0)	28.9 (0.6)	102.2 (4.6)	5.2 (0.2)
Second quartile (4.55%–5.90% unemployed)	204.6 (11.9)	10.7 (0.3)	553.8 (27.6)	29.0 (0.9)	102.5 (6.3)	5.4 (0.4)
Third quartile (5.90%–7.74% unemployed)	198.6 (12.2)	10.7 (0.3)	537.9 (35.3)	28.9 (1.3)	103.4 (8.3)	5.6 (0.5)
Highest quartile (>7.74% unemployed)	188.0 (11.9)	10.6 (0.3)	511.9 (42.2)	29.0 (1.5)	103.0 (9.8)	5.8 (0.6)
Level of reductions to working age benefits per person per year**†**						
Lowest quartile (<£321.5)	209.2 (12.1)	10.7 (0.3)	561.8 (23.0)	28.8 (0.6)	103.3 (5.5)	5.3 (0.3)
Second quartile (£321.5–£403)	205.4 (11.8)	10.7 (0.3)	556.8 (25.6)	29.0 (0.9)	102.7 (7.3)	5.4 (0.4)
Third quartile (£403–£479)	196.5 (13.2)	10.6 (0.3)	532.9 (38.3)	28.8 (1.3)	104.2 (8.7)	5.7 (0.6)
Highest quartile (>£479)	190.7 (14.3)	10.7 (0.3)	517.9 (46.5)	29.1 (1.3)	101.0 (8.2)	5.7 (0.6)
IMD**‡**						
1 (most deprived)	187.7 (14.0)	10.7 (0.4)	505.6 (47.0)	28.7 (1.6)	103.4 (10.7)	5.9 (0.7)
2	196.3 (12.9)	10.7 (0.3)	535.3 (36.1)	29.1 (1.3)	102.5 (8.3)	5.6 (0.5)
3	201.5 (12.1)	10.7 (0.3)	550.3 (28.1)	29.1 (0.9)	102.4 (6.8)	5.4 (0.4)
4	207.1 (11.7)	10.7 (0.3)	558.1 (27.1)	29.0 (0.8)	102.0 (6.6)	5.3 (0.4)
5 (least deprived)	210.0 (11.4)	10.7 (0.2)	562.9 (20.2)	28.7 (0.6)	103.9 (5.0)	5.3 (0.2)
Rural/urban area						
Predominantly urban	194.7 (14.5)	10.6 (0.3)	523.6 (40.2)	28.6 (1.3)	104.9 (9.0)	5.8 (0.5)
Urban with significant rural	205.2 (11.8)	10.7 (0.2)	559.2 (19.7)	29.2 (0.6)	101.4 (4.3)	5.3 (0.2)
Predominantly rural	209.4 (10.9)	10.8 (0.3)	570.5 (17.2)	29.4 (0.5)	99.6 (4.4)	5.1 (0.2)

*GDHI categorised into four quartiles.

†We stratified by quartiles of reductions in working age benefit by LA, using a dataset estimating the cumulative decreases in benefits for working age people due to welfare reforms between 2010 and 2015 for each LA.

‡IMD represents relative deprivation of LAs, categorised into quintiles.

GDHI, gross disposable household income; HFSS, high in fat, sugar and salt; IMD, Index of Multiple Deprivation; LA, local authority.

**Figure 3:**
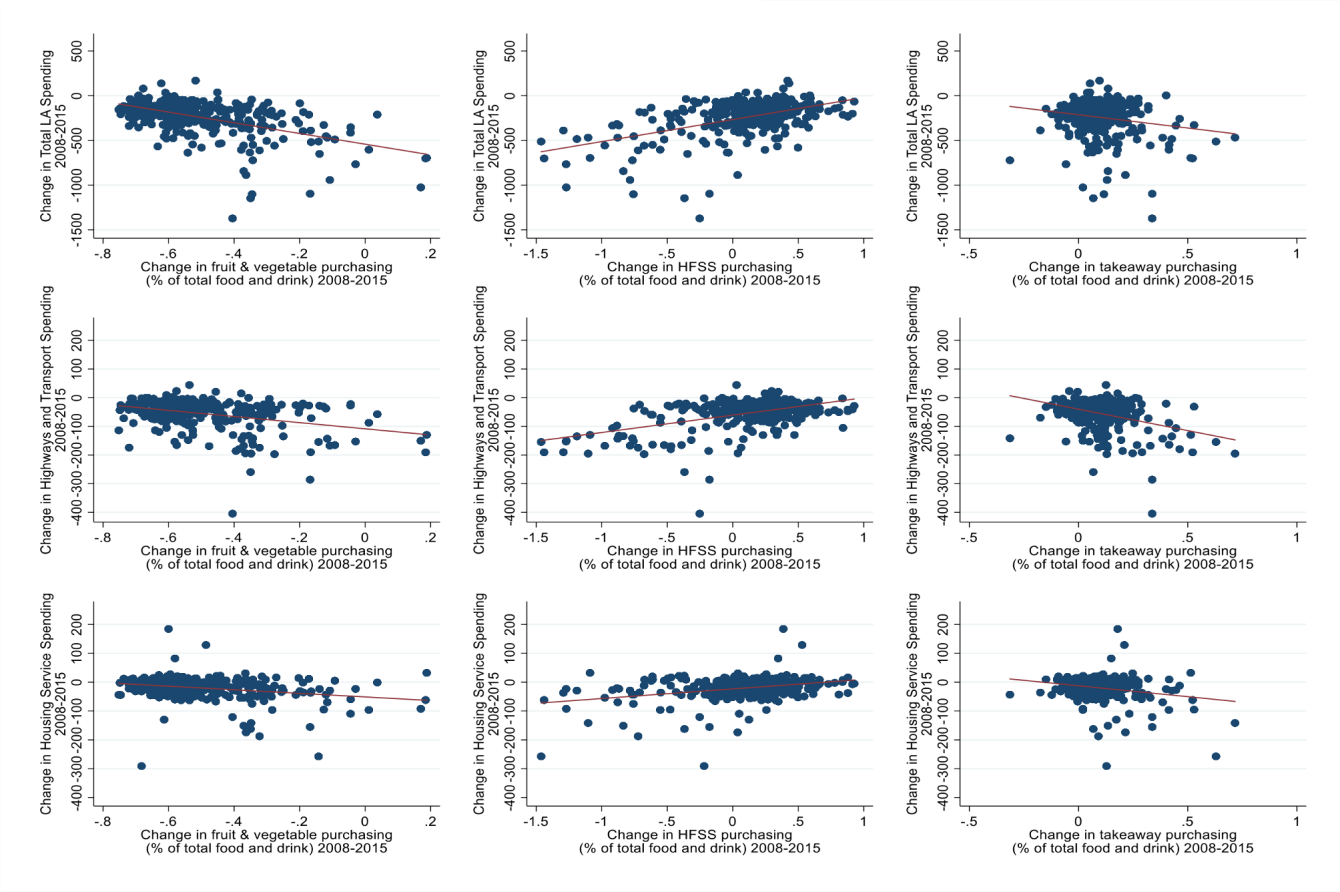
Change in LA spending in relation to changes in food spending. HFSS, high in fat, sugar and salt; LA, local authority.

Figure 3 shows the change in LA spending (2008–2015) plotted with changes in food purchasing (2008–2015). The x axes represent the change in food purchasing as a percentage of total food and drink purchasing between 2008 and 2015 and the y axes show change in LA spending between 2008 and 2015. The top row shows change in total LA spending, the middle row highways and transport spending, and the bottom row housing spending. These graphs show that larger decreases in LA spending were associated with smaller decreases in fruit and vegetable purchasing and larger increases in takeaway purchasing. Larger decreases in LA spending were also related to larger decreases in HFSS purchasing between 2008 and 2015.

### Fixed effects analysis of association between spending and food purchasing as a percentage of food and drink expenditure

#### Total LA spending

A 10% decrease in total LA service spending was associated with a decrease in HFSS as a percentage of overall food and drink purchasing (−0.078, 95% CI −0.152 to –0.056)) (table 3). Our results were similar following adjustment for GDHI and unemployment rate (−0.071, 95% CI −0.093 to –0.050)), representing an increase of 0.07 percentage points. This persisted in the most deprived LAs (−0.072, 95% CI −0.113 to –0.033 for quintile 1 and −0.088, 95% CI −0.126 to –0.040 for quintile 2). There was a greater effect urban areas (−0.080, 95% CI −0.107 to –0.052) compared with rural (−0.030, 95% CI −0.056 to –0.004). This relationship was statistically significant in the higher quartiles of level of working age benefit reductions (eg, quintile 4: −0.075, 95% CI −0.116 to –0.034), with a larger effect size than the lowest quartile, where this relationship was not statistically significant (−0.035, 95% CI −0.082 to 0.012).

**Table 3:** Impact of total LA spending on food purchasing

	Fruit and vegetables	HFSS foods	Takeaways
	Purchasing as a percentage of total food and drink purchasing (%)	Purchasing as a percentage of total food and drink purchasing (%)	Purchasing as a percentage of total food and drink purchasing (%)
Unadjusted model			
Full sample	0.006 (–0.002 to 0.014) p=0.154	−0.078 (–0.152 to –0.056) p<0.001	0.017 (0.008 to 0.026) p<0.001
Adjusted model*			
Full Sample	0.007 (–0.002 to 0.015) p=0.110	−0.071 (–0.093 to –0.050) p<0.001	0.015 (0.006 to 0.024) p=0.001
Adjusted model stratified by IMD†			
1 (most deprived)	−0.003 (–0.022 to 0.015) p=0.735	−0.072 (–0.113 to –0.033) p=0.001	0.025 (–0.000 to 0.049) p=0.050
2	0.020 (–0.001 to 0.041) p=0.059	−0.088 (–0.126 to –0.040) p=0.001	0.021 (0.002 to 0.040) p=0.028
3	−0.006 (–0.020 to 0.009) p=0.448	−0.024 (–0.079 to 0.021) p=0.291	0.003 (–0.014 to 0.015) p=0.972
4	0.006 (–0.012 to 0.023) p=0.528	−0.053 (–0.102 to –0.004) p=0.034	0.012 (–0.007 to 0.031) p=0.200
5 (least deprived)	−0.006 (–0.017 to 0.005) p=0.250	−0.048 (–0.097 to 0.001) p=0.054	0.022 (0.008 to 0.035) p=0.002
Adjusted model stratified by rural/urban area			
Predominantly Urban	0.002 (–0.010 to 0.013) p=0.788	−0.080 (–0.107 to –0.052) p<0.001	0.024 (0.011 to 0.036) p<0.001
Urban with significant rural	0.005 (–0.007 to 0.015) p=0.416	0.005 (–0.028 to 0.039) p=0.746	0.003 (–0.009 to 0.014) p=0.630
Predominantly rural	0.004 (–0.005 to 0.014) p=0.354	−0.030 (–0.056 to –0.004) p=0.026	−0.006 (–0.018 to 0.006) p=0.320
Adjusted model stratified by level of reductions in working age benefits‡			
Lowest quartile (<£321.5)	0.001 (–0.015 to 0.018) p=0.885	−0.035 (–0.082 to 0.012) p=0.142	0.016 (0.001 to 0.031) p=0.037
Second quartile (£321.5–£403)	0.001 (–0.012 to 0.013) p=0.891	−0.047 (–0.085 to –0.010) p=0.014	0.011 (–0.004 to 0.025) p=0.161
Third quartile (£403–£479)	−0.005 (–0.022 to 0.012) p=0.572	−0.079 (–0.119 to –0.039) p<0.001	0.020 (0.002 to 0.039) p=0.032
Highest quartile (>£479)	0.008 (–0.009 to 0.025) p=0.369	−0.075 (–0.116 to –0.034) p<0.001	0.028 (0.009 to 0.047) p=0.005

The coefficients represent the percentage point change in purchasing with a 10% decrease in LA service spending (95% CIs in brackets).

*Model adjusted for GDHI and unemployment rate.

†IMD represents relative deprivation of LAs, categorised into quintiles.

‡We stratified by quartiles of reductions in working age benefit by LA, using a dataset estimating the cumulative decreases in benefits for working age people due to welfare reforms between 2010 and 2015 for each LA.

GDHI, gross disposable household income; HFSS, high in fat, sugar and salt; IMD, Index of Multiple Deprivation; LA, local authority.

A 10% decrease in total LA spending was associated with a 0.017 percentage point increase in takeaway purchasing (95% CI 0.008 to 0.026). This persisted following adjustment (0.015, 95% CI 0.006 to 0.024). Our results suggest effect modification by rural/urban area status (urban: 0.024 95% CI 0.011 to 0.036; rural: −0.006, 95% CI −0.018 to 0.006). A stronger relationship was seen for LAs with greatest reductions in working age benefits, but there was little effect modification by IMD. We did not identify an association between total LA spending and fruit and vegetable purchasing (0.006, 95% CI −0.002 to 0.0014 (unadjusted model)).

#### Highways and transport spending

A 10% decrease in highways and transport expenditure was associated with a 0.006 percentage point decrease in fruit and vegetable purchasing as a percentage of total food and drink purchasing (95% CI −0.009 to –0.002) in both the unadjusted and adjusted models (table 4). This association was only statistically significant in urban areas (−0.007, 95% CI −0.012 to –0.001) when stratified. A 10% decrease in highways and transport expenditure was associated with a 0.011 percentage point decrease in purchasing of HFSS foods (95% CI −0.020 to –0.002), but this was no longer reached statistical significance after adjustment for study covariates (−0.005, 95% CI −0.0013 to 0.003). A 10% decrease in LA highways and transport spending was associated with a 0.006 percentage point increase in takeaways (95% CI 0.002 to 0.011). There was evidence of effect modification by rural/urban area status (urban: 0.007, 95% CI 0.001 to 0.014; rural: −0.001, 95% CI −0.006 to 0.009). There was little evidence of effect modification by reductions in working age benefits or IMD for highways and transport spending, except for HFSS foods.

**Table 4:** Impact of highways and transport spending on food purchasing

	Fruit and vegetables	HFSS foods	Takeaways
	Purchasing as a percentage of total food and drink purchasing (%)	Purchasing as a percentage of total food and drink purchasing (%)	Purchasing as a percentage of total food and drink purchasing (%)
Unadjusted model			
Full sample	−0.006 (−0.009 to –0.002) p=0.002	−0.011 (−0.020 to –0.002) p=0.017	0.006 (0.002 to 0.011) p=0.003
Adjusted model*			
Full sample	−0.006 (−0.009 to –0.002) p=0.002	−0.005 (−0.013 to 0.003) p=0.223	0.006 (0.001 to 0.010) p=0.010
Adjusted model stratified by IMD†			
1 (most deprived)	−0.009 (−0.019 to 0.001) p=0.057	−0.007 (−0.025 to 0.011) p=0.442	0.010 (−0.001 to 0.022) p=0.078
2	−0.004 (−0.011 to 0.002) p=0.193	−0.002 (−0.017 to 0.013) p=0.800	0.007 (−0.004 to 0.017) p=0.183
3	−0.002 (−0.009 to 0.005) p=0.599	0.005 (−0.013 to 0.024) p=0.561	0.002 (−0.006. 0.010) p=0.566
4	−0.003 (−0.009 to 0.004) p=0.413	0.006 (−0.012 to 0.023) p=0.515	0.004 (−0.004 to 0.012) p=0.362
5 (least deprived)	−0.005 (−0.012 to 0.003) p=0.255	−0.007 (−0.030 to 0.015) p=0.520	0.001 (−0.006 to 0.008) p=0.845
Adjusted model stratified by rural/urban area			
Predominantly urban	−0.007 (−0.012 to –0.001) p=0.013	−0.008 (−0.020 to 0.004) p=0.211	0.007 (0.001 to 0.014) p=0.024
Urban with significant rural	0.002 (−0.006 to 0.009) p=0.688	−0.006 (−0.025 to 0.014) p=0.556	−0.003 (−0.008 to 0.003) p=0.421
Predominantly rural	−0.002 (−0.006 to 0.002) p=0.261	0.006 (−0.005 to 0.018) p=0.295	−0.001 (−0.006 to 0.009) p=0.658
Adjusted model stratified by level of reductions in working age benefits‡			
Lowest quartile (<£321.5)	−0.006 (−0.014 to 0.003) p=0.197	0.006 (−0.018 to 0.029) p=0.619	0.001 (−0.006 to 0.009) p=0.704
Second quartile (£321.5−£403)	−0.004 (−0.009 to 0.001) p=0.088	0.006 (−0.006 to 0.019) p=0.327	0.003 (−0.004 to 0.010) p=0.357
Third quartile (£403−£479)	−0.007 (−0.015 to 0.001) p=0.066	−0.025 (−0.031 to 0.002) p=0.080	0.011 (0.002 to 0.021) p=0.023
Highest quartile (>£479)	−0.005 (−0.011 to 0.002) p=0.181	−0.002 (−0.018 to 0.013) p=0.762	0.006 (−0.003 to 0.015) p=0.180

The coefficients represent the percentage point change in purchasing with a 10% decrease in LA service spending (95% CIs in brackets).

*Model adjusted for GDHI, unemployment rate and LA expenditure on other services.

†IMD represents relative deprivation of LAs, categorised into quintiles.

‡We stratified by quartiles of reductions in working age benefit by LA, using a dataset estimating the cumulative decreases in benefits for working age people due to welfare reforms between 2010 and 2015 for each LA.

GDHI, gross disposable household income; HFSS, high in fat, sugar and salt; IMD, Index of Multiple Deprivation; LA, local authority.

#### Housing service spending

A 10% reduction in LA housing service spending was associated with a small decrease in HFSS purchasing in unadjusted (−0.005, 95% CI −0.009 to –0.001) and adjusted models (−0.006, 95% CI −0.010 to –0.002) (table 5). Although not statistically significant, housing spending and HFSS had the opposite relationship in the most deprived quintile, suggesting potential effect modification (0.004, 95% CI −0.010 to 0.018). There was little evidence of effect modification by level of reductions in working age benefits. Housing service spending was not statistically significantly associated with change in fruit and vegetables (0.001, 95% CI −0.000 to 0.003) nor takeaway purchasing (0.000, 95% CI −0.001 to 0.001).

**Table 5:** Impact of housing service expenditure on food purchasing

	Fruit and vegetables	HFSS foods	Takeaways
	Purchasing as a percentage of total food and drink purchasing (%)	Purchasing as a percentage of total food and drink purchasing (%)	Purchasing as a percentage of total food and drink purchasing (%)
Unadjusted model			
Full sample	0.001 (−0.000 to 0.003) p=0.125	−0.005 (−0.009 to –0.001) p=0.013	0.000 (−0.001 to 0.001) p=0.961
Adjusted model*			
Full Sample	0.001 (−0.000 to 0.003) p=0.119	−0.006 (−0.010 to –0.002) p=0.001	0.000 (−0.001 to 0.002) p=0.676
Adjusted model stratified by IMD†			
1 (most deprived)	0.000 (−0.004 to 0.004) p=0.881	0.004 (−0.010 to 0.018) p=0.604	−0.001 (−0.005 to 0.003) p=0.684
2	0.003 (−0.001 to 0.007) p=0.056	−0.011 (−0.019 to –0.004) p=0.004	−0.000 (−0.003 to 0.003) p=0.931
3	−0.000 (−0.004 to 0.003) p=0.824	−0.012 (−0.022 to –0.003) p=0.009	0.003 (−0.001 to 0.007) p=0.115
4	−0.001 (−0.005 to 0.003) p=0.602	−0.003 (−0.010 to 0.005) p=0.504	0.001 (−0.002 to 0.003) p=0.651
5 (least deprived)	−0.001 (−0.004 to 0.002) p=0.593	0.000 (−0.007 to 0.007) p=0.933	0.000 (−0.002 to 0.003) p=0.874
Adjusted model stratified by rural/urban area			
Predominantly urban	−0.000 (−0.002 to 0.003) p=0.841	−0.005 (−0.011 to 0.001) p=0.136	−0.000 (−0.002 to 0.003) p=0.769
Urban with significant rural	0.003 (−0.001 to 0.006) p=0.117	−0.008 (−0.015 to –0.001) p=0.035	0.001 (−0.001 to 0.002) p=0.595
Predominantly rural	0.002 (−0.000 to 0.003) p=0.054	−0.005 (−0.009 to –0.000) p=0.049	−0.000 (−0.002 to 0.002) p=0.702
Adjusted model stratified by level of reductions in working age benefits‡			
Lowest quartile (<£321.5)	−0.002 (−0.005 to 0.001) p=0.189	−0.001 (−0.008 to 0.006) p=0.721	0.001 (−0.002 to 0.003) p=0.689
Second quartile (£321.5–£403)	0.001 (−0.002 to 0.004) p=0.401	−0.004 (−0.010 to 0.002) p=0.150	0.002 (−0.001 to 0.004) p=0.214
Third quartile (£403–£479)	−0.000 (−0.004 to 0.003) p=0.822	−0.017 (−0.026 to –0.010) p<0.001	0.003 (−0.001 to 0.006) p=0.094
Highest quartile (>£479)	0.002 (−0.001 to 0.006) p=0.152	0.000 (−0.011 to 0.011) p=0.973	−0.002 (−0.006 to 0.001) p=0.204

The coefficients represent the percentage point change in purchasing with a 10% decrease in LA service spending (95% CIs in brackets).

*Model adjusted for GDHI, unemployment rate and LA expenditure on other services.

†IMD represents relative deprivation of LAs categorised into quintiles.

‡We stratified by quartiles of reductions in working age benefit by LA, using a dataset estimating the cumulative decreases in benefits for working age people due to welfare reforms between 2010 and 2015 for each LA.

GDHI, gross disposable household income; HFSS, high in fat, sugar and salt; IMD, Index of Multiple Deprivation; LA, local authority.

### Fixed effects analysis of association between spending and absolute food purchasing

With regards to absolute purchasing, a decrease in total LA spending was associated with a 28p decrease in money spent on fruit and vegetables per year and a £1.96 decrease in HFSS foods per year (online supplemental appendix 2). A decrease in highways and transport spending was associated with a 9p increase in money spent on takeaways (online supplemental appendix 3). A decrease in highways and transport expenditure was associated with a 42p increase in purchasing of HFSS foods in rural areas only, but this relationship was not significant for the full sample. A decrease in housing expenditure was associated with a 15p increase in money spent on fruit and vegetables per year, a 20p increase for HFSS foods and a 6p increase for takeaways (online supplemental appendix 4).

10.1136/bmjnph-2021-000346.supp2 Supplementary data

10.1136/bmjnph-2021-000346.supp3 Supplementary data

10.1136/bmjnph-2021-000346.supp4 Supplementary data

### Additional analyses

Our negative exposure control analysis (online supplemental appendix 5) found that LA cultural spend was not associated with food and drink purchasing for the full sample (eg, fruit and vegetables: −0.001, 95% CI −0.003 to 0.0.002). Three of the 42 associations tested were statistically significant. These were seen in the middle quartiles or quintiles when stratified by IMD and level of reductions in working age benefits (eg, fourth quintile of IMD for takeaways: −0.008, 95% CI −0.014 to –0.003)). Our examination of medium-term effects using time lags demonstrated that effects over time may be complex—these analyses produced a range of results which differed across outcomes and time periods, with effect directions differing depending on the time lag (online supplemental appendix 6). A consistent effect direction was only seen for total LA spending and HFSS foods, with a decrease in total LA spending being associated with a decrease in HFSS foods with no lag and one and two year lags. The analysis with bootstrapping had little impact on the results, with small changes to p values and CIs (online supplemental appendix 7). When total spending was used as the exposure, slight increases in the p values and widening of the confidence intervals were seen, while with highways and transport as the exposure, p values slightly decreased and some confidence intervals slightly narrowed.

10.1136/bmjnph-2021-000346.supp5 Supplementary data

10.1136/bmjnph-2021-000346.supp6 Supplementary data

10.1136/bmjnph-2021-000346.supp7 Supplementary data

## Discussion

Our study provides evidence that changes in LA spending were associated with impacts on food purchasing at an LA population level. We found that a decrease in total LA spending was associated with a decrease in HFSS food purchasing and an increase in takeaway purchasing as a percentage of total food and drink purchasing in the same year. Decreases in highways and transport spending were associated with small decreases in fruit and vegetables purchasing and small increases in takeaway purchasing as a percentage of total food and drink purchasing in the same year. These relationships were apparent only in urban areas when stratified, suggesting effect modification by urban/rural area status. A decrease in housing spending was associated with a small decrease in HFSS food purchasing as a percentage of total food and drink purchasing in the same year.

We found a 17% reduction in LA service spending (2008–2015). This aligns with findings by the National Audit Office that LA service spending decreased by 19% in real terms between 2010–2011 and 2017–2018.^8^ In our fixed effects models, we found that a 10% decrease in total LA service spending was associated with a 0.07 percentage point decrease in HFSS food purchasing and a 0.02 percentage point increase in takeaway purchasing. These results suggest a potential substitution away from HFSS foods towards takeaways, as we saw decreases in HFSS foods alongside increases (or null effects) in takeaways. The effects sizes are small, relating to spending changes of less than £2 per year for HFSS foods and less than 9p for takeaways (online supplemental appendices 3 and 4). However, evidence suggests that impacts of reductions to LA service expenditures are intersectional and concentrated in certain groups.^2 42^ As our results represent impacts at the LA level, it is likely that these effects are heterogeneous at the individual level and impacts may be larger for certain groups - individual-level research is needed to disentangle these effects further. Additionally, our effect estimates for total LA service expenditures were larger than the results when highways and transport and housing expenditure were examined individually. This suggests that the associations between total LA spending and HFSS and takeaways described may only be partially driven by transport and housing expenditure, which we had hypothesised as the most likely pathways. This leaves unanswered questions about additional pathways.

One potential pathway is highways and transport expenditure. We found that the relationship between transport spending and food purchasing was only statistically significant in urban areas. Urban areas tend to have more areas without good access to supermarkets and other types of food store which can make accessing affordable healthy food difficult.^43^ Urban dwellers have lower car ownership and thus a higher percentage of people would be affected by changes to public transport.^44^ Furthermore, convenience stores and small supermarkets, which are often frequent in urban areas, are more likely to have lower availability and quality of healthful foods including fruit and vegetables and higher prices.^18^ Takeaway outlets are also higher in urban areas.^45^ These provide potential pathways linking reductions in LA transport spending and a shift away from fresh fruit and vegetable purchasing towards takeaway purchasing.^43^

Furthermore, decreases in housing service expenditure were associated with small decreases in the percentage of money spent on HFSS in overall food and drink budgets. Variation in LA spending due to different priorities has been described in the literature, with particularly large variation in spending on housing services.^30 42^ However, impacts of different LA strategies on housing are not clear.^46^ LAs experiencing greater overall cuts tended to reduce housing expenditure less than those with lower cuts, and thus these results may be reflective of lower cuts to other services, although we adjusted for expenditure on other types of services.^8^ Given that the highest levels of LA reductions tended to occur in more deprived areas where need for housing expenditure is greater, there may be effect modification by IMD and level of reductions to working age benefit. Indeed, we found that in the most deprived LAs, a decrease in LA spending was associated with a small increase in HFSS purchasing, contrary to the decreases in HFSS seen in quintiles 2–5. Interestingly, the percentage of fruit and vegetables and takeaways in total food and drink budgets did not change, only HFSS. We would have expected that if resources are tight, more expensive products (ie, fruits and vegetables) would decrease before or in addition to cheaper HFSS foods.^22^ It is possible that cuts to housing service were concentrated in areas of service spending that impact relatively small groups of more vulnerable people, and thus did not directly impact financial resources of a large enough number of households to show an effect on purchasing of these foods. Furthermore, decreases in housing expenditure were associated with small increases in money spent on fruit and vegetables, HFSS, and takeaways (online supplemental appendix 4). This may suggest an overall increase in food and drink spending where HFSS increased the least, and thus decreased as a percentage of spending, contrary to our hypothesis. Short term economic shocks have been reported to lead to increases in individuals’ food expenditure in the short term,^47^ and it is possible that a similar impact is being seen here.

This research has several strengths. To the best of our knowledge, this is the first study to investigate the impact of reductions in LA spending due to UK austerity measures on food purchasing. Our study was undertaken using publicly available data. We used a large panel dataset for 324 LAs in England between 2008 and 2015. We used fixed effects linear models to account for time-invariant factors and adjusted for several time-varying factors. Our analysis using bootstrapping methods suggested that effects were not down to a small number of LAs (online supplemental appendix 7). This study also has limitations. Issues with data quality may be present as administrative data may be subject to bias from missing data, miscoding, and misclassification. Non-response from specific groups such as low-income households to the Living Costs and Food Survey (which was the basis of the LA-level estimates we used) may also be present and lead to an underestimation of impacts, as austerity measures have been suggested to have been regressive.^32 42^ However, the Living Costs and Food Survey is weighted to compensate for non-response, and the data we used were developed using a method which took into account employment, ethnicity, population and income characteristics.^32^ Furthermore, as this study found very small coefficients, these may be due to random noise rather than evidencing a genuine effect – further research is needed to examine the impacts of LA spending on food purchasing and consumption. We conducted a negative exposure control analysis, where three of the 42 associations tested were statistically significant (online supplemental appendix 5). This small number of statistically significant associations may be the result of multiple comparisons and thus our negative exposure control analysis may suggest limited residual confounding. Conversely, this may suggest some confounding, particularly for the middle quintiles/quartiles of IMD and level of benefit reductions. Another option is that this may suggest that LAs with greatest reductions in cultural services also reduced other services the most. A key aspect of austerity policies is welfare reform and benefit changes. However, LA data on benefit spending are not accurate due to differential impacts on working age people and the elderly, and changes in benefits (eg, switches from LA-reported Job Seekers Allowance to regionally reported Universal Credit), not allowing us to investigate benefit spending as an exposure. However, we were able to stratify by quartile of average reductions to working age benefits by LA – although this approach has limitations due to approaching a time-varying variable as a time-invariant variable, it enabled us to examine how reductions in LA spending interact with impacts of welfare reform. Our examination of medium-term effects using lagged analysis gave complex results and suggested the need for further research to investigate these effects (online supplemental appendix 6). As this study is ecological, causality cannot be inferred; nevertheless, this research is an important first step in filling the evidence gap in this area.

In conclusion, our panel study reports that changes in LA service spending may have small impacts on food purchasing. We are the first to examine impacts of austerity policies on diets, as research has hitherto focused on impacts of welfare reform on food insecurity, and thus, this work represents an advance in this field. Our findings suggest that LA spending is an important avenue for future research regarding impacts of public spending on dietary and health outcomes, especially as this research has raised questions regarding pathways in addition to highways and transport and housing service spending. We consistently found decreases in LA service spending to be associated with decreases in purchasing of foods HFSS as a percentage of food and drink expenditure, which may confer positive health impacts. However, we also found that a decrease in LA service expenditure may lead to a decrease in fruit and vegetable expenditure and an increase in takeaway expenditure, and thus negative impacts on peoples’ health may also arise. Although the associations we have described are small at an individual level, there may be larger impacts in some segments of the population, and small shifts in dietary patterns can potentially have large population health impacts.^12^ Further individual-level research is needed to disentangle these potential impacts and elucidate potential mechanisms. Policy-makers and healthcare workers should consider the diet and health impacts of reductions to LA budgets, as our results may suggest that changes to LA service spending affect the foods that people buy.

## Data Availability

Data are available on reasonable request. All data are publicly available and are available on reasonable request.
