# Supplementary material for: Changes to local area public sector spending and food purchasing in England: a longitudinal ecological study
Source: BMJ Nutr Prev Health. 2022 Mar 4;5(1):72–86. doi: 10.1136/bmjnph-2021-000346 (PMC9237904; doi:10.1136/bmjnph-2021-000346)
Supplement: Supplementary data [file bmjnph-2021-000346supp001.pdf]

Appendix 1: Product categories included in outcome variables

Variable	Product categories included
Fresh fruits and vegetables	Citrus fruits, bananas, apples, pears, stone fruits, berries, other fresh, chilled or frozen fruits, leaf and stem vegetables, cabbages, vegetables grown for their fruit (eg. cucumber, peppers, peas), root crops (non-starchy eg. carrots, tomatoes, onions) and potatoes.
HFSS foods	Soft drinks, cakes, chocolate confectionary, sugar confectionary, ice cream, morning goods (ie. pastries), puddings, sweet biscuits, breakfast cereals, yoghurts, sweetened milk-based drinks, sweetened juices, pizza, ready meals, meal centres including breaded and battered products, crisps and savoury snacks and chips and potato products
Takeaways	Chicken from chip shop/KFC, meat pies and pasties, fish and fish products from fish and chip shop, burgers, kebabs, Indian and Chinese, pizza, and takeaway cakes, pastries, buns and biscuits, takeaway sandwiches, confectionary, ice cream, and crisps from takeaway.
