# Supplementary material for: Changes to local area public sector spending and food purchasing in England: a longitudinal ecological study
Source: BMJ Nutr Prev Health. 2022 Mar 4;5(1):72–86. doi: 10.1136/bmjnph-2021-000346 (PMC9237904; doi:10.1136/bmjnph-2021-000346)
Supplement: Supplementary data [file bmjnph-2021-000346supp002.pdf]

Appendix 2: Impact of Total LA Spending on Food Purchasing (£ per year). The coefficients represent the change in purchasing in pounds and pence with a 10% decrease in LA service spending (95% Confidence Intervals in brackets).

| | Fruit and Vegetables | HFSS Foods | Takeaways |
|---|---|---|---|
| Unadjusted model | | | |
| Full Sample | -0.271 (-0.531, -0.095) p=0.042 | -1.995 (-2.758, -1.232) p<0.001 | 0.060 (-0.127, 0.247) p=0.529 |
| Adjusted Model[1] | | | |
| Full Sample | -0.278 (-0.540, -0.017) p=0.037 | -1.958 (-2.724, -1.192) p<0.001 | 0.016 (-0.169, 0.007) p=0.866 |
| Adjusted Model stratified by IMD[2] | | | |
| 1 (most deprived) | -0.310 (-0.752, 0.131) p=0.165 | -1.502 (-2.864, -0.140) p=0.031 | 0.191 (-0.264, 0.646) p=0.405 |
| 2 | -0.595 (-1.269, 0.079) p=0.082 | -2.586 (-4.529, -0.642) p=0.010 | 0.024 (0.388, 0.436) p=0.908 |
| 3 | -0.457 (-1.129, 0.216) p=0.180 | -1.497 (-3.253, 0.259) p=0.093 | -0.244 (-0.597, 0.109) p=0.172 |
| 4 | -0.526 (-1.104, 0.051) p=0.073 | -2.235 (-4.104, 0.367) p=0.020 | -0.074 (-0.428, 0.280) p=0.678 |
| 5 (least deprived) | -0.305 (-0.935, 0.325) p=0.337 | -1.522 (-3.079, 0.034) p=0.055 | 0.197 (-0.160, 0.553) p=0.275 |
| Adjusted Model stratified by rural/urban area | | | |
| Predominantly Urban | -0.246 (-0.588, 0.096) p=0.158 | -1.498 (-2.458, -0.538) p=0.002 | 0.213 (-0.030, 0.455) p=0.085 |
| Urban with Significant Rural | -0.220 (-0.770, 0.331) p=0.427 | -0.847 (-2.435, 0.740) p=0.289 | -0.072 (-0.417, 0.273) p=0.677 |
| Predominantly Rural | -0.159 (-0.579, 0.261) p=0.453 | -1.352 (-2.495, -0.209) p=0.021 | -0.242 (-0.523, 0.039) p=0.091 |
| Adjusted Model stratified by level of reductions in working age benefits[3] | | | |
| Lowest quartile (<£321.5) | -0.564 (-1.133, 0.006) p=0.052 | -2.167 (-3.790, 0.545) p=0.010 | -0.050 (-0.434, 0.336) p=0.797 |
| Second quartile (£321.5 - £403) | -0.359 (-0.915, 0.197) p=0.202 | -1.504 (-3.010, 0.002) p=0.050 | 0.012 (-0.264, 0.287) p=0.934 |
| Third quartile (£403 - £479) | -0.685 (-1.305, -0.065) p=0.031 | -2.259 (-3.901, 0.616) p=0.008 | 0.031 (-0.317, 0.379) p=0.860 |
| Highest quartile (>£479) | -0.123 (-0.531, 0.285) p=0.549 | -1.448 (-2.871, 0.025) p=0.046 | 0.286 (-0.076, 0.648) p=0.120 |

[1] Model adjusted for GDHI and unemployment rate

[2] IMD represents relative deprivation of LAs, categorised into quintiles.

[3] We stratified by quartiles of reductions in working age benefit by LA, using a dataset estimating the cumulative decreases in benefits for working age people due to welfare reforms between 2010-2015 for each LA.
