# Supplementary material for: Changes to local area public sector spending and food purchasing in England: a longitudinal ecological study
Source: BMJ Nutr Prev Health. 2022 Mar 4;5(1):72–86. doi: 10.1136/bmjnph-2021-000346 (PMC9237904; doi:10.1136/bmjnph-2021-000346)
Supplement: Supplementary data [file bmjnph-2021-000346supp003.pdf]

Appendix 3: Impact of Highways and Transport Spending on Food Purchasing (£ per year). The coefficients represent the change in purchasing in pounds and pence with a 10% decrease in LA service spending (95% Confidence Intervals in brackets).

	Fruit and Vegetables	HFSS Foods	Takeaways
Unadjusted model			
Full Sample	-0.057 (-0.181, 0.067) p=0.369	-0.101 (-0.419, 0.217) p=0.532	0.111 (0.034, 0.189) p=0.005
Adjusted Model[1]			
Full Sample	-0.061 (-0.186, 0.063) p=0.333	-0.031 (-0.339, 0.277) p=0.844	0.087 (0.009, 0.164) p=0.029
Adjusted Model stratified by IMD[2]			
1 (most deprived)	-0.120 (-0.445, 0.015) p=0.067	-0.165 (-0.836, 0.508) p=0.626	0.129 (-0.055, 0.313) p=0.166
2	-0.387 (-0.622, -0.153) p=0.002	-0.639 (-1.162, -0.115) p=0.018	-0.057 (-0.214, 0.100) p=0.472
3	0.239 (-0.020, 0.497) p=0.070	0.673 (-0.043, 1.389) p=0.065	0.174 (0.012, 0.337) p=0.036
4	-0.178 (-0.460, 0.105) p=-0.213	-0.359 (-1.161, 0.442) p=0.374	-0.015 (-0.187, 0.158) p=0.867
5 (least deprived)	0.228 (-0.088, 0.545) p=0.154	0.557 (-0.196, 1.310) p=0.144	0.138 (-0.060, 0.336) p=0.170
Adjusted Model stratified by rural/urban area			
Predominantly Urban	-0.145 (-0.321, 0.032) p=0.109	-0.210 (-0.630, 0.209) p=0.324	0.094 (-0.009, 0.197) p=0.075
Urban with Significant Rural	0.083 (-0.243, 0.409) p=0.613	-0.042 (-0.994, 0.911) p=0.930	-0.015 (-0.228, 0.197) p=0.885
Predominantly Rural	0.104 (-0.097, 0.305) p=0.307	0.418 (0.005, 0.831) p=0.047	0.024 (-0.082, 0.130) p=0.652
Adjusted Model stratified by level of reductions in working age benefits[3]			
Lowest quartile (<£321.5)	-0.036 (-0.339, 0.266) p=0.811	0.153 (-0.664, 0.970) p=0.711	0.028 (-0.172, 0.228) p=0.781
Second quartile (£321.5 - £403)	0.180 (-0.042, 0.401) p=0.111	0.668 (0.117, 1.220) p=0.018	0.140 (-0.010, 0.290) p=0.067
Third quartile (£403 - £479)	-0.293 (-0.559, -0.026) p=0.032	-0.617 (-1.252, 0.019) p=0.057	0.121 (-0.026, 0.267) p=0.104
Highest quartile (>£479)	-0.073 (-0.150, 0.004) p=0.063	-0.190 (-0.719, 0.339) p=0.477	0.044 (-0.098, 0.186) p=0.538

[1] Model adjusted for GDHI, unemployment rate, and LA expenditure on other services.

[2] IMD represents relative deprivation of LAs, categorised into quintiles.

[3] We stratified by quartiles of reductions in working age benefit by LA, using a dataset estimating the cumulative decreases in benefits for working age people due to welfare reforms between 2010-2015 for each LA.
