# Supplementary material for: Changes to local area public sector spending and food purchasing in England: a longitudinal ecological study
Source: BMJ Nutr Prev Health. 2022 Mar 4;5(1):72–86. doi: 10.1136/bmjnph-2021-000346 (PMC9237904; doi:10.1136/bmjnph-2021-000346)
Supplement: Supplementary data [file bmjnph-2021-000346supp004.pdf]

Appendix 4: Impact of Housing Service Expenditure on Food Purchasing (£ per year). The coefficients represent the change in purchasing in pounds and pence with a 10% decrease in LA service spending (95% Confidence Intervals in brackets).

| | Fruit and Vegetables | HFSS Foods | Takeaways |
|---|---|---|---|
| Unadjusted model | | | |
| Full Sample | 0.149 (0.100, 0.199) p<0.001 | 0.220 (0.069, 0.371) p=0.004 | 0.056 (0.012, 0.090) p=0.001 |
| Adjusted Model[1] | | | |
| Full Sample | 0.145 (0.097, 0.193) p<0.001 | 0.204 (0.058, 0.349) p=0.006 | 0.056 (0.022, 0.089) p=0.001 |
| Adjusted Model stratified by IMD[2] | | | |
| 1 (most deprived) | 0.207 (0.094, 0.319) p<0.001 | 0.511 (0.067, 0.955) p=0.025 | 0.062 (-0.013, 0.136) p=0.102 |
| 2 | 0.170 (0.062, 0.279) p=0.003 | 0.110 (-0.227, 0.447) p=0.516 | 0.069 (-0.017, 0.156) p=0.114 |
| 3 | 0.097 (-0.935, 0.230) p=0.146 | 0.086 (-0.297. 0.469) p=0.656 | 0.100 (0.020, 0.179) p=0.014 |
| 4 | 0.079 (-0.018, 0.176) p=0.109 | 0.239 (-0.141, 0.619) p=0.214 | 0.066 (-0.014, 0.145) p=0.104 |
| 5 (least deprived) | 0.034 (-0.051, 0.119) p=0.430 | 0.126 (-0.111, 0.363) p=0.293 | 0.004 (-0.051, 0.059) p=0.886 |
| Adjusted Model stratified by rural/urban area | | | |
| Predominantly Urban | 0.289 (0.116, 0.272) p<0.001 | 0.345 (0.130, 0.560) p=0.002 | 0.081 (0.034, 0.128) p=0.001 |
| Urban with Significant Rural | 0.063 (-0.004, 0.129) p=0.063 | -0.061 (-0.322, 0.200) p=0.641 | 0.011 (-0.041, 0.064) p=0.665 |
| Predominantly Rural | 0.120 (0.034, 0.203) p=0.005 | 0.174 (-0.032, 0.380) p=0.098 | 0.041 (-0.017, 0.099) p=0.161 |
| Adjusted Model stratified by level of reductions in working age benefits[3] | | | |
| Lowest quartile (<£321.5) | 0.015 (-0.069, 0.099) p=0.729 | 0.062 (-0.180, 0.303) p=0.613 | 0.005 (-0.055, 0.065) p=0.873 |
| Second quartile (£321.5 - £403) | 0.087 (-0.007, 0.175) p=0.052 | 0.141 (-0.152, 0.435) p=0.341 | 0.068 (-0.002, 0.137) p=0.056 |
| Third quartile (£403 - £479) | 0.190 (0.073, 0.306) p=0.002 | 0.218 (-0.134, 0.569) p=0.222 | 0.131 (0.051, 0.211) p=0.002 |
| Highest quartile (>£479) | 0.196 (0.087, 0.306) p=0.001 | 0.367 (0.010, 0.743) p=0.056 | 0.033 (-0.029, 0.095) p=0.297 |

[1] Model adjusted for GDHI, unemployment rate, and LA expenditure on other services.

[2] IMD represents relative deprivation of LAs, categorised into quintiles.

[3] We stratified by quartiles of reductions in working age benefit by LA, using a dataset estimating the cumulative decreases in benefits for working age people due to welfare reforms between 2010-2015 for each LA.
