# Supplementary material for: Changes to local area public sector spending and food purchasing in England: a longitudinal ecological study
Source: BMJ Nutr Prev Health. 2022 Mar 4;5(1):72–86. doi: 10.1136/bmjnph-2021-000346 (PMC9237904; doi:10.1136/bmjnph-2021-000346)
Supplement: Supplementary data [file bmjnph-2021-000346supp005.pdf]

Appendix 5: Negative Control Analysis of impact of LA Cultural Spend on food purchasing. The coefficients represent the percentage point change in purchasing with a 10% decrease in LA service spending (95% Confidence Intervals in brackets).

	Fruit and Vegetables	HFSS Foods	Takeaways
	Purchasing as a percentage of total food and drink expenditure (%)	Purchasing as a percentage of total food and drink expenditure (%)	Purchasing as a percentage of total food and drink expenditure (%)
Unadjusted model			
Full Sample	-0.001 (-0.003, 0.002) p=0.508	0.003 (-0.003, 0.010) p=0.316	-0.003 (-0.006, 0.001) p=0.165
Adjusted Model[1]			
Full Sample	-0.001 (-0.003, 0.002) p=0.518	0.006 (-0.001, 0.013) p=0.111	-0.003 (-0.007, 0.001) p=0.105
Adjusted Model stratified by IMD[2]			
1 (most deprived)	-0.005 (-0.017, 0.007) p=0.382	0.011 (-0.014, 0.037) p=0.378	-0.013 (-0.026, 0.000) p=0.050
2	0.000 (-0.003, 0.003) p=0.950	0.006 (-0.004, 0.017) p=0.219	-0.002 (-0.007, 0.003) p=0.499
3	-0.008 (-0.015, -0.001) p=0.023	0.014 (-0.002, 0.013) p=0.078	0.002 (-0.005, 0.009) p=0.518
4	0.003 (-0.001, 0.013) p=0.218	0.006 (-0.019, 0.023) p=0.503	-0.008 (-0.014, -0.003) p=0.004
5 (least deprived)	-0.000 (-0.007, 0.006) p=0.917	-0.011 (-0.028, 0.006) p=0.215	0.002 (-0.004, 0.007) p=0.587
Adjusted Model stratified by rural/urban area			
Predominantly Urban	0.000 (-0.003, 0.003) p=0.942	0.009 (-0.002, 0.020) p=0.114	-0.004 (-0.008, 0.001) p=0.107
Urban with Significant Rural	-0.002 (-0.009, 0.005) p=0.656	-0.005 (-0.033, 0.023) p=0.714	-0.003 (-0.006, 0.005) p=0.911
Predominantly Rural	-0.001 (-0.004, 0.003) p=0.642	-0.008 (-0.019, 0.003) p=0.154	-0.001 (-0.005, 0.003) p=0.648
Adjusted Model stratified by level of reductions in working age benefits[3]			
Lowest quartile (<£321.5)	0.001 (-0.005, 0.007) p=0.738	0.001 (-0.016, 0.013) p=0.851	-0.001 (-0.006, 0.005) p=0.847
Second quartile (£321.5 - £403)	-0.001 (-0.008, 0.006) p=0.713	-0.000 (-0.015, 0.015) p=0.985	0.001 (-0.006, 0.007) p=0.887
Third quartile (£403 - £479)	-0.003 (-0.013, 0.006) p=0.482	0.030 (0.005, 0.055) p=0.017	-0.010 (-0.021, 0.001) p=0.063
Highest quartile (>£479)	-0.000 (-0.003, 0.003) p=0.893	0.003 (-0.005, 0.011) p=0.421	-0.004 (-0.011, 0.002) p=0.209

[1] Model adjusted for GDHI, unemployment rate, and LA expenditure on other services.

[2] IMD represents relative deprivation of LAs, categorised into quintiles.

[3] We stratified by quartiles of reductions in working age benefit by LA, using a dataset estimating the cumulative decreases in benefits for working age people due to welfare reforms between 2010-2015 for each LA.
