# Supplementary material for: Changes to local area public sector spending and food purchasing in England: a longitudinal ecological study
Source: BMJ Nutr Prev Health. 2022 Mar 4;5(1):72–86. doi: 10.1136/bmjnph-2021-000346 (PMC9237904; doi:10.1136/bmjnph-2021-000346)
Supplement: Supplementary data [file bmjnph-2021-000346supp006.pdf]

Appendix 6: Adjusted models with lag effects (no lag, one year lag, two year lag). The coefficients represent the percentage point change in purchasing with a 10% decrease in LA service spending (95% Confidence Intervals in brackets).

	Fruit and Vegetables	HFSS Foods	Takeaways
	Purchasing as a percentage of total food and drink expenditure (%)	Purchasing as a percentage of total food and drink expenditure (%)	Purchasing as a percentage of total food and drink expenditure (%)
Total LA spending[1]			
No lag	-0.002 (-0.014, 0.010) p=0.734	-0.037 (-0.057, 0.017) p<0.001	0.003 (-0.009, 0.014) p=0.658
Lagged one year	0.009 (0.000, 0.018) p=0.038	-0.024 (0.045, -0.003) p=0.026	-0.003 (-0.016, 0.009) p=0.608
Lagged two years	0.003 (-0.008, 0.013) p=0.607	-0.028 (0.050, 0.006) p=0.011	0.010 (-0.003, 0.022) p=0.122
Highways and transport spending[2]			
No lag	-0.012 (-0.018 -0.007) p<0.001	-0.002 (-0.012, 0.008) p=0.645	0.009 (0.238, 0.015) p=0.006
Lagged one year	0.009 (0.003, 0.013) p<0.001	0.001 (-0.007, 0.010) p=0.757	-0.012 (-0.020, -0.005) p=0.001
Lagged two years	-0.003 (-0.007, 0.002) p=0.219	-0.009 (-0.018, -0.000) p=0.048	0.012 (0.006, 0.018) p<0.001
Housing Spending[2]			
No lag	0.000 (-0.002, 0.002) p=0.623	-0.003 (-0.008, 0.001) p=0.120	0.001 (-0.001, 0.002) p=0.561
Lagged one year	0.001 (-0.006, 0.003) p=0.157	0.003 (-0.001, 0.007) p=0.131	-0.002 (-0.004, -0.000) p=0.036
Lagged two years	-0.002 (-0.003, 0.000) p=0.078	-0.001 (-0.005, 0.003) p=0.508	0.000 (-0.001, 0.002) p=0.619

[1] Model adjusted for GDHI and unemployment rate.

[2] Model adjusted for GDHI, unemployment rate, and LA expenditure on other services.
