# Supplementary material for: Changes to local area public sector spending and food purchasing in England: a longitudinal ecological study
Source: BMJ Nutr Prev Health. 2022 Mar 4;5(1):72–86. doi: 10.1136/bmjnph-2021-000346 (PMC9237904; doi:10.1136/bmjnph-2021-000346)
Supplement: Supplementary data [file bmjnph-2021-000346supp007.pdf]

Appendix 7: Impact of Total LA Spending on Food Purchasing with bootstrapped coefficients. The coefficients represent the percentage point change in purchasing with a 10% decrease in LA service spending (95% Confidence Intervals in brackets).

	Fruit and Vegetables	HFSS Foods	Takeaways
	Purchasing as a percentage of total food and drink expenditure (%)	Purchasing as a percentage of total food and drink expenditure (%)	Purchasing as a percentage of total food and drink expenditure (%)
Total LA spending			
Unadjusted model	0.006 (-0.003, 0.015) p=0.208	-0.078 (-0.097, -0.060) p<0.001	0.017 (0.006, 0.027) p=0.002
Adjusted model[1]	0.007 (-0.002, 0.016) p=0.141	-0.071 (-0.090, -0.053) p<0.001	0.015 (0.005, 0.025) p=0.004
Highways and transport spending			
Unadjusted model	-0.006 (-0.009, -0.002) p=0.001	-0.011 (-0.019, -0.003) p=0.008	0.006 (0.003, 0.010) p=0.001
Adjusted model[2]	-0.005 (-0.009, -0.002) p=0.001	-0.005 (-0.013, 0.002) p=0.183	0.006 (0.002, 0.009) p=0.005
Housing spending			
Unadjusted model	0.001 (-0.000, 0.003) p=0.123	-0.005 (-0.008, -0.001) p=0.005	0.000 (-0.001, 0.002) p=0.963
Adjusted model[2]	0.001 (-0.000, 0.003) p=0.125	-0.006 (-0.009, -0.003) p<0.001	0.000 (-0.001, 0.002) p=0.697

[1] Model adjusted for GDHI and unemployment rate.

[2] Model adjusted for GDHI, unemployment rate, and LA expenditure on other services.
